# Phylogenetic lineages of tuberculosis isolates and their association with patient demographics in Tanzania

**DOI:** 10.1186/s12864-022-08791-3

**Published:** 2022-08-05

**Authors:** Beatrice Kemilembe Mutayoba, Michael Hoelscher, Norbert Heinrich, Moses L. Joloba, Eligius Lyamuya, Andrew Martin Kilale, Nyagosya Segere Range, Bernard James Ngowi, Nyanda Elias Ntinginya, Saidi Mwinjuma Mfaume, Amani Wilfred, Basra Doulla, Johnson Lyimo, Riziki Kisonga, Amri Kingalu, Jupiter Marina Kabahita, Ocung Guido, Joel Kabugo, Isa Adam, Moses Luutu, Maria Magdalene Namaganda, Joanitah Namutebi, George William Kasule, Hasfah Nakato, Henry Byabajungu, Pius Lutaaya, Kenneth Musisi, Denis Oola, Gerald Mboowa, Michel Pletschette

**Affiliations:** 1 Department of Preventive Services, Ministry of Health, Dodoma, Tanzania; 2 Department of Infectious Diseases and Tropical Medicine, Medical Center of the, University of Munich, Munich, Germany; 3 National Tuberculosis Reference Laboratory/Supranational Reference Laboratory, Luzira, Uganda; 4 Department of Immunology and Molecular Biology, College of Health Sciences, Makerere University, Kampala, Uganda; 5 Department of Microbiology and Immunology, Muhimbili University of Health and Allied Sciences (MUHAS), Dar es Salaam, Tanzania; 6 Muhimbili Research Centre, National Institute for Medical Research (NIMR), Dar es Salaam, Tanzania; 7 University of Dar Es Salaam, Mbeya College of Health and Allied Sciences, Mbeya, Tanzania; 8 Mbeya Medical Research Centre, NIMR, Mbeya, Tanzania; 9 Central Tuberculosis Reference Laboratory, Ministry of Health, National TB and Leprosy Programme, Dar es Salaam, Tanzania; 10 Department of Preventive Services, Ministry of Health, National Tuberculosis and Leprosy Programme, Dodoma, Tanzania; 11 Africa Centres for Disease Control and Prevention, African Union Commission, Addis Ababa, Ethiopia

**Keywords:** Phylogenetic, Lineages, Mycobacterial isolates, Whole-genome sequencing, Tanzania

## Abstract

**Background:**

*Mycobacterium tuberculosis* presents several lineages each with distinct characteristics of evolutionary status, transmissibility, drug resistance, host interaction, latency, and vaccine efficacy. Whole genome sequencing (WGS) has emerged as a new diagnostic tool to reliably inform the occurrence of phylogenetic lineages of *Mycobacterium tuberculosis* and examine their relationship with patient demographic characteristics and multidrug-resistance development.

**Methods:**

191 *Mycobacterium tuberculosis* isolates obtained from a 2017/2018 Tanzanian drug resistance survey were sequenced on the Illumina Miseq platform at Supranational Tuberculosis Reference Laboratory in Uganda. Obtained fast-q files were imported into tools for resistance profiling and lineage inference (Kvarq v0.12.2, Mykrobe v0.8.1 and TBprofiler v3.0.5). Additionally for phylogenetic tree construction, RaxML-NG v1.0.3(25) was used to generate a maximum likelihood phylogeny with 800 bootstrap replicates. The resulting trees were plotted, annotated and visualized using ggtree v2.0.4

**Results:**

Most [172(90.0%)] of the isolates were from newly treated Pulmonary TB patients. Coinfection with HIV was observed in 33(17.3%) TB patients. Of the 191 isolates, 22(11.5%) were resistant to one or more commonly used first line anti-TB drugs (FLD), 9(4.7%) isolates were MDR-TB while 3(1.6%) were resistant to all the drugs. Of the 24 isolates with any resistance conferring mutations, 13(54.2%) and 10(41.6%) had mutations in genes associated with resistance to INH and RIF respectively. The findings also show four major lineages i.e. Lineage 3[81 (42.4%)], followed by Lineage 4 [74 (38.7%)], the Lineage 1 [23 (12.0%)] and Lineages 2 [13 (6.8%)] circulaing in Tanzania.

**Conclusion:**

The findings in this study show that Lineage 3 is the most prevalent lineage in Tanzania whereas drug resistant mutations were more frequent among isolates that belonged to Lineage 4.

**Supplementary Information:**

The online version contains supplementary material available at 10.1186/s12864-022-08791-3.

## Background

Collective tuberculosis (TB) drug resistance analysis studies from Sub-Saharan African countries report the prevalence of multi-drug resistant tuberculosis (DR-TB) in new cases to be 2.1%. This low prevalence is however likely to be due to under reporting and lack of intensive access to drug resistance testing (DST) [1]. Phylogenetic analysis has been revolutionary in understanding the evolutionary development and diversification of pathogenic organisms and is useful in understanding their distribution. Seven major lineages of *Mycobacterium tuberculosis (M. tuberculosis)*, have been globally documented each exhibiting distinct characteristics from another in terms of evolutionary status, transmissibility, drug resistance, host interaction, latency, and vaccine efficacy [2]. These major lineages have been further subdivided into sub-lineages for example lineage 2 (East Asian) and lineage 4 (Euro-American) comprise the Beijing and Haarlem genotypes respectively. These show variation in virulence and pathogenicity with high association for tuberculosis outbreaks and drug-resistance [3]. Understanding TB transmission is key in disease control and prevention and the later highly depends upon rapid case detection. Rapid case detection should incorporate timely accurate drug susceptibility testing (DST) of *Mycobacterium tuberculosis* (*M. tuberculosis*) isolates. Several testing methods have been endorsed by the World Health Organisation (WHO) to test and confirm *M. tuberculosis*, revealing its phenotypic and genotypic characteristics. The most widely used phenotypic method i.e., culture and drug susceptibility testing are notoriously challenging and require stringent biosafety requirements to obtain the actual diagnosis [4]. These conventional methods are slow for comprehensive understanding of the *M. tuberculosis* infections to administer appropriate treatment. The molecular methods which include line-probe assays (LPAs) and Xpert MTB/RIF assay (Cepheid, Sunnyvale, CA, USA) tend to overcome some of these challenges but fall short on covering the entire genomic understanding of the *M. tuberculosis* strains [5]. New molecular diagnostic methods based on genomic DNA sequencing have increasingly expounded TB genomics characteristically describing phylogeny of *M. tuberculosis* [6]. These include IS6110-RFLP methodology necessitating Southern blotting, spoligotyping, mycobacterial interspersed repetitive and whole genome sequencing (WGS) [7–10]. These have greatly improved the understanding of detection of unsuspected transmission and discrimination between re-infection, relapse and phylogeographical variations of the *M. tuberculosis* [11, 12].

Tanzania ranks among the seven TB high burden countries worldwide [13] with a total of 75,845 cases notified and incidence of 253 per 100,000 in 2018. The regional distribution of the cases in the country ranks Dar es Salaam city as the major contributor of TB cases notification at 20% contribution of all cases [13] with the rest in other regions of Mwanza, Arusha, Geita, Dodoma, Manyara and Mbeya but less has been done to understand the phylogenetic distribution.

Worldwide, vast numbers of sequences of *M. tuberculosis* strains have been generated with several libraries of single nucleotide poly-morphisms (SNPs) and other variants generated for comparative purposes. The research in low- and middle-income countries where Tanzania falls still lags in this area and more work needs to be done to guide accurate clinical decisions and provide more evidence of the prevailing strains in the country. To comprehensively understand the phylogeographical variations in Tanzania, we performed WGS on the drug resistance survey (DRS) isolates sourced all around Tanzania. Findings from this work should inform intervention strategies and future MDR-TB monitoring tactics. The sequence data will also help to understand the genomic characteristics of *M. tuberculosis* isolates and their resistant mutations prevalent among pulmonary TB patients enrolled during the second national anti-TB drug resistance survey in Tanzania.

## Methods

### Study design, population and sampling

This was a cross sectional national drug resistance survey conducted from June 2017 to July 2018. A cluster sampling strategy was used and the unit of sampling was a diagnostic center that notified 8 and more smear positive cases in 2015. Based on this, a total of 45 clusters were selected and in each cluster, a total of 34 new smear positive pulmonary TB patients and all previously treated smear positive pulmonary TB cases diagnosed during the intake period for the survey were enrolled. Sputum samples were collected and forwarded to the Central TB Reference Laboratory (CTRL) in Dar es Salaam for smear microscopy, culture, strain identification and susceptibility testing following standard NTLP procedures. For WGS, a total of 627 culture positive isolates were shipped to the National TB Reference Laboratory/Supranational Tuberculosis Reference Laboratory- Uganda.

### Sub-culture and DNA extraction for whole-genome sequencing

All isolates were sub-cultured on selective Middlebrook 7H11 agar (Becton and Dickson, USA), incubated at 37^0^C in a CO_2_ incubator (Panasonic, Osaka, Japan) and monitored weekly for growth. Once sufficient bacterial colonies were observed, these were harvested into a 15 ml Falcon tube with 1.0 ml of sterile water, followed by a thirty-minute heat inactivation at 85^0^C. High quality genomic DNA was extracted using an in-house cetyltrimethylammonium bromide (CTAB) method previously described [14]. Integrity of the extracted DNA was assessed using the TapeStation 4150 (Agilent USA) with the Agilent Genomic DNA ScreenTape and reagents. Purity of the bacterial DNA was assessed using the NanoDrop 2000c (ThermoFisher Scientific).

### Library preparation and sequencing

Genomic libraries were prepared using the Illumina Nextera XT library preparation kit following manufacturer’s instructions [15]. Quality of the prepared libraries was assessed with the Agilent 4150 using the D1000 High sensitivity ScreenTape and reagents. Libraries were sequenced on the MiSeq (Illumina, San Diego, CA, USA) using the Illumina MiSeq V3 cartridge at the Supranational Tuberculosis Reference Laboratory in Uganda.

### Bioinformatics analysis

#### Resistance and lineage determination

A total of 191 samples were sequenced. Quality of reads was assessed using FastQC [16] v0.11.8 and MultiQC [17] v1.0. Bad quality bases were trimmed off using Trimmomatic v0.39 [18]. Three tools for resistance profiling and lineage inference namely Kvarq [19] v0.12.2, Mykrobe [20] v0.8.1 and TBprofiler [21] v3.0.5 were run.

#### Phylogenetic tree construction

*De-novo* genome assembly of all samples was done using Unicycler v0.4.8[22]. The assembled genomes were then annotated using Prokka [23] to generate genomic feature files to be used as input for Roary v3.13.0 [24] which was then used to generate a core gene multiple sequence alignment. Using the GTR + G substitution model, a maximum likelihood phylogeny was constructed using RaxML-NG v1.0.3 [25] with 800 bootstrap replicates with H37Rv reference strain NC_000962.3 as the reference and *Mycobacterium canettii* NC_015848.1 as the out-group. The resulting trees were plotted, annotated and visualized using ggtree v2.0.4 [26].

#### Ethical considerations

The study was approved by the National Health Research Ethics Committee of Tanzania and the Department of Infectious Diseases and Tropical Medicine, Medical Center of the University of Munich, Munich, Germany. Written informed consent or assent was obtained from all participants.

## Results

### Demographic characteristics of TB patients from whom the isolates were collected

Of the 627 samples received at the NTRL-Uganda, 10 were rejected and only 617 were sub-cultured. Of these 265 (43%) yielded either no growth (negative), contaminated or NTM and could not be processed further for WGS. Of the 352 samples that yielded a positive TB culture, 191 (54%) were sequenced due to resource constraints. Of these, 133 (70.0%) were from male TB patients. The mean age (standard deviation) of the TB patients from whom the isolates were collected was 37.5 (± 13.8) years. Most (107; 55.8%) of the TB patients were aged 25–44 years. Most [169 (88.0%)] of the isolates were from newly treated pulmonary TB patients. Coinfection with HIV was observed in 33 (17.3%) of the 191 TB patients. Of the 191 isolates, 22 (11.5%) were resistant to one or more commonly used first line anti-TB drugs (FLD). While 3 (1.6%) were resistant to all the drugs, 9 (4.7%) isolates were MDR-TB (Supplementary data Table 1).

**Table 1:** Patients’ history of previous TB treatment and HIV status by *M. tuberculosis* lineages

*M. tuberculosis* lineages	Total n (%)	History of TB, n (%)		HIV Status, n (%)	
		Previously treated	Newly treated	HIV positive	HIV negative
**Lineage 2**	13 (6.8)	1(5.3)	12 (7.0)	1 (3.0)	12 (7.6)
**Lineage 3**	81 (42.4)	9 (47.4)	72 (41.9)	15 (45.5)	66 (41.8)
**Lineage 1**	23 (12.0)	2 (10.5)	21 (12.1)	2 (6.1)	21 (13.3)
**Lineage 4**	74 (38.7)	7 (36.8)	67 (39.0)	15 (45.5)	59 (37.3)
**Total**	**191**	**19 (10.0)**	**172 (90.0)**	**33 (17.3)**	**158 (82.7)**

#### Phylogenetic analysis

From the 191 M*. tuberculosis* isolates, four main lineages were identified at different frequencies (Table 1). The dominant lineage was Lineage 3 [81 (42.4%)], followed by Lineage 4 [74 (38.7%)], then Lineage 1 [23 (1209%)] and Lineage 2 [13 (6.8%)] (Table 1). Lineage 3 was the most prevalent among isolates from previously treated TB cases 9 (47.4%) as compared to 72 (41.9%) among isolates from newly treated patients. Lineage 4 dominated 7 (36.8%) those previously treated as compared to 67 (39.0%) of the newly treated. Lineage 1 was reported in 2 (10.5%) of the previously treated as compared to 21 (12.1%) of the newly treated patients. Lineage 2 was isolated in 1 (5.3%) of the previously treated TB case while the newly treated patients harboured 12 (6.9%) of these isolates. Lineage 3 was the most prevalent in both HIV positive 15 (5.5%) and HIV negative 66 (41.8%). This was also the case for Lineage 4 with 59 (37.3%) isolates from HIV negative and 15 (45.5%) from HIV positive patients (Table 1).

#### M*. tuberculosis* Lineages and their correlation with drug resistance conferring mutation

**Fig. 1:**
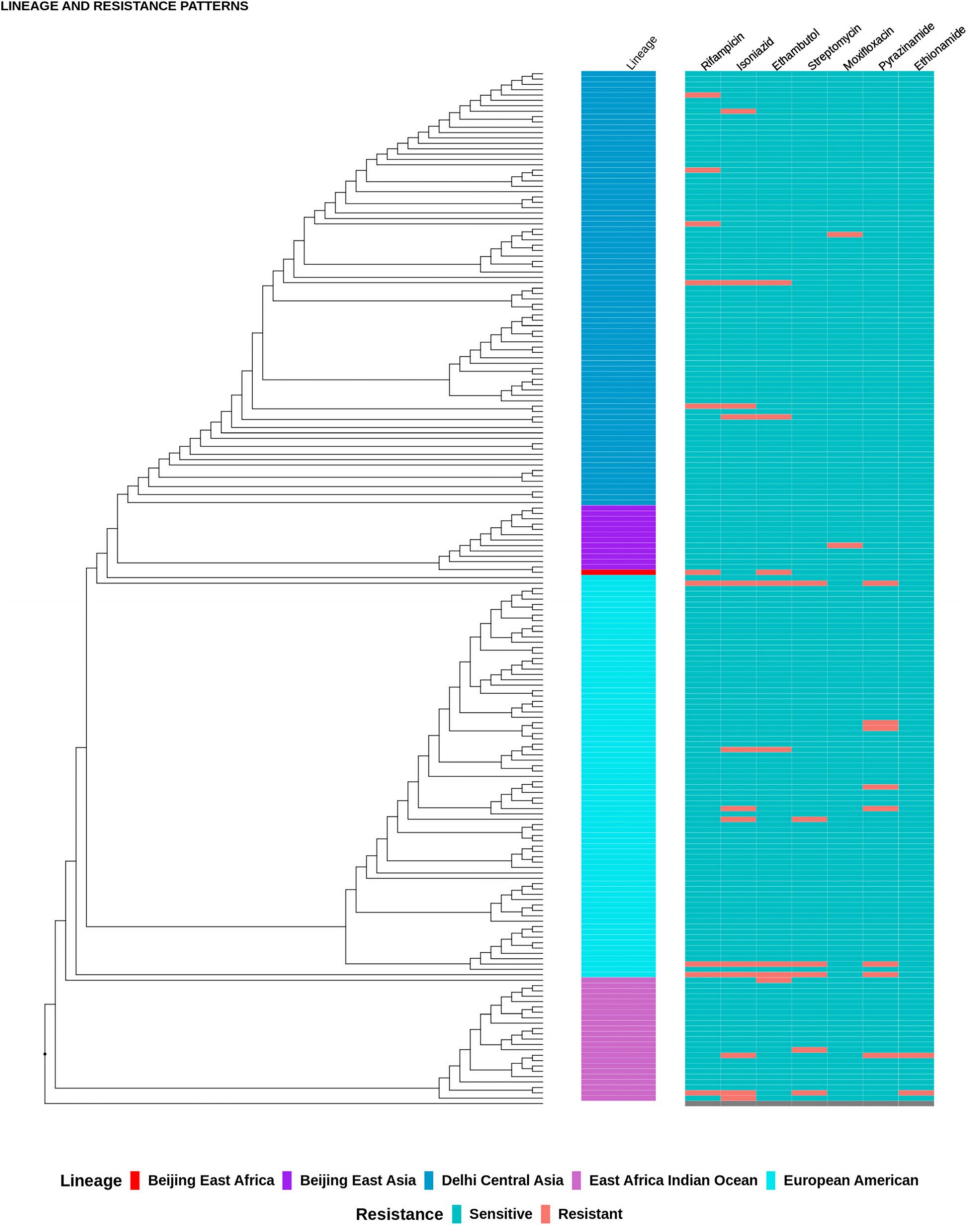
Phylogenetic tree showing association between Mycobacterium tuberculosis lineages and drug resistance

While the Lineage 2 had 1 (7.7%) isolate that showed resistance to rifampicin and ethambutol, Lineage 3 had 7 (8.6%) isolates resistant to FLDs, out of which 3 (3.7%) were MDR-TB. For Lineage 1, out of the 23 isolates, 5 (21.7%) were resistant to FLDs and 2 (8.7%) were MDR-TB. Out of 74 isolates for Lineage 4, 9 (12.2%) were resistant to FLDs and 3 (4.1%) were MDR-TB (Table 2, Fig. 1 and Supplementary data Table 2).

**Table 2:** Anti-TB drug resistance stratified by M. tuberculosis lineages, *N* = 191

*M. tuberculosis* lineages	Total n	Anti-TB drugs resistance (row %)					
		INH n (%)	RMP n (%)	EMB n (%)	PZA n (%)	MDR n (%)	RFLD^a^ n (%)
**Lineage 2**	13	0 (0)	1 (7.7)	1 (7.7)	0 (0)	1 (7.7)	1 (7.7)
**Lineage 3**	81	4 (4.9)	5 (6.1)	2 (2.4)	0 (0)	3 (3.7)	7 (8.6)
**Lineage 1**	23	3 (13.0)	1 (4.4)	1 (4.4)	1 (4.4)	2 (8.7)	5 (21.7)
**Lineage 4**	74	6 (8.1)	3 (4.1)	4 (5.4)	7 (9.5)	3 (4.1)	9 (12.2)

*RFLD*^a^ Resistant to first line drugs, *INH* Isoniazid, *RMP* Rifampicin, *EMB* Ethambutol, *PZA* Pyrazinamide, *MDR* Multi-drug resistance

#### Frequency of drug resistant mutations

The most prevalent Isoniazid conferring mutation was *KatG.*Ser315Thr [9 (37.5%)]. The *inhA.*Ser94Ala and *fabG1 c.-15C* > *T*, *c.-8 T* > *A*, CTG607CTA had 1 (4.2%) mutation each. All Isoniazid conferring mutations were classified as common with a high resistance level observed in *fabG1 c.-15C* > *T* and *KatG*. *Ser315Thr* while the promoter regions of *inhA.*Ser94Ala, *fabG1.*CTG607CTA and *fabG1 c.-8 T* > *A*. All had a low detected resistance level (Table 3).

**Table 3:** Frequency of drug resistance mutations, *N* = 24

Drug	Gene	Mutation	Resistant (n/N (%))	Classification of the mutation	Resistance level
**Isoniazid**	fabG1	c.-15C > T	1/24 (4.2)	Common	High
	fabG1	CTG607CTA	1/24 (4.2)	Common	Low
	fabG1	c.-8 T > A	1/24 (4.2)	Common	Low
	inhA	Ser94Ala	1/24 (4.2)	Common	Low
	katG	Ser315Thr	9/24 (37.5)	Common	High
**Rifampicin**	rpoB	Gln432Glu	3/24 (12.5)	Common	High
	rpoB	His445Asn	1/24 (4.2)	Rare	Low
	rpoB	Leu430Pro	1/24 (4.2)	Common	High
	rpoB	Ser441Gln	2/24 (8.3)	Rare	Low
	rpoB	Ser450Leu	3/24 (12.5)	Common	High
**Ethambutol**	embB	Asp1024Asn	1/24 (4.2)	Common	High
	embB	Gln497Arg	2/24 (8.4)	Common	High
	embB	Leu359Ile	1/24 (4.2)	Common	High
	embB	Met306Ile	4/24 (16.7)	Common	High
**Pyrazinamide**	pncA	Ala30Val	2/24 (8.4)	Common	High
	pncA	E111$	1/24 (4.2)	Common	Low
	pncA	GAG331TAG	2/24 (8.4)	Common	High
	pncA	Leu172Pro	1/24 (4.2)	Common	High
	pncA	Phe106Leu	1/24 (4.2)	Rare	Low
	pncA	Thr160Ala	1/24 (4.2)	Common	High
**Streptomycin**	gid	Pro93Leu	1/24 (4.2)	-	
	rpsL	Lys88Met	4/24 (16.7)	-	
	rrs	Ser172Cys	1/24 (4.2)	-	
**Ethionamide**	fabG	c.-15C > T	1/24 (4.2)	-	
	inhA	Ser94Ala	1/24 (4.2)	-	
**Fluoroquinolones**	gyrA	Ala90Val	1/24 (4.2)	Common (LEV, MOX CC)	
	gyrA	Asp94Gly	1/24 (4.2)	Common	

*Note*: Indicates missing information on classification

The most prevalent Rifampicin resistance-conferring mutation were *rpoB.*Gln432Glu and *rpoB.*Ser450Leu with each accounting for a total of 3 (12.5%), while the remaining mutations were as follows: *rpoB.*Ser441Gln was found twice (8.3%), *rpoB.*His445Asn 1 (4.2%), and *rpoB.Leu430Pro* as well only 1 (4.2%). Rifampicin resistance-conferring mutation *rpoB.*His445Asn and *rpoB.*Ser441Gln were classified as rare with an equally low observed resistance level, while *rpoB.*Gln432Glu, *rpoB.*Leu430Pro and *rpoB.*Ser450Leu were classified as commonly occurring mutation with a high resistance level observed (Table 3).

Resistance-conferring mutations to Ethambutol in the *embCAB* loci were found in 8 (33.3%) isolates, with *embB.*Met306Ile being the most prevalent in 4 (16.7%), followed by *embB.*Gln497Arg at 2 (8.4%) while *embB.*Asp1024Asn and *embB.*Leu359Ile each had 1 (4.2%) mutation prevalence. All Ethambutol driven mutations were classified as common with a high resistance level. Resistance to Pyrazinamide at the *pncA* locus was identified in 8(33.3%) isolates and none with *rpsA*. The most prevalent Pyrazinamide resistance-conferring mutation *pncA.*Ala30Val and GAG331TAG with each accounting for 2 (8.4%), while the remaining mutations of *pncA.*Leu172Pro, *pncA.*Phe106Leu, *pncA.*Thr160Ala*,* and *pncA.*E111$ all had 1 (4.2%) mutation each. Resistance conferring mutation at pncA.Phe106Leu was classified as rare while *pncA.*Leu172Pro, *pncA.*Ala30Val, *pncA.*GAG331TAG, *pncA.*Thr160Ala and *pncA.*E111 were considered common (Table 3).

For Streptomycin resistance, mutation in the *rspL.*Lys88Met was reported at 4 (16.7%) and were more frequent followed by resistance-conferring mutation in *rrs*. *Ser172Cys* at 1 (4.2%) while mutations in the *gidB* promoter region of *Pro93Leu* accounted for 1 (4.2%). Resistance to Ethionamide due to mutations in *fabG1* and *inhA* were found in 2(8.3%) of the isolates. Resistance-conferring mutation at loci *fabG1 c.-15C* > *T* and *inhA.*Ser94Ala each Ser94Ala were each reported at 1 (4.2%). Mutations in the conserved quinolone resistance-determining region (QRDR) of *gyrA* at position *Ala90Val* at 1 (4.2%) as well as *Asp94Gly* at 1 (4.2%) and classified as common (Table 3 and Supplementary data Table 3).

## Discussion

This study reports the existence of heterogeneity among MTBC lineages circulating in Tanzania. Central Asian Lineage (L3) was the most predominant followed by Euro American (L4), Indo-Oceanic (L1) and East-Asian [2] lineage respectively. This is contrary to an earlier study done in the same setting that reported L4 to be the more widely distributed lineage as compared to L3 [27]. Previous studies have also highlighted that the East Asian lineage has only been recently circulating within the African continent which is consistent to findings in this study [28]. Furthermore, L3 was reported to be widely distributing among the newly treated population in this study as compared to the population with a previous history of TB treatment which may be suggestive of a high TB transmission pattern of the widely transmitting L3 in Tanzania.

In this study, we show that East Asian lineage and Euro American lineages were largely found in TB patients living with HIV. This is a rare finding in Tanzania since no previous study has demonstrated no such association between TB drug resistance and HIV infection [29, 30]. However, our findings are in line with the findings from a recent study conducted in Haiti that reported the same MTB lineages harbouring MDR-TB resistance patterns as well as the higher risk of MDR-TB infection in people living with HIV (PLHIV) [31].

Although previous treatment for TB is the strongest risk factor for development of DR-TB [32–35], treatment-naïve patients may also acquire drug resistance due to either transmission of resistant strains or spontaneous mutations. In our study we report strains resistant to some SLDs which are not being used to treat TB in Tanzania. However, similar findings were reported in a study conducted in India to determine the antimicrobial susceptibility to first-line and second line anti-TB drug resistance among newly diagnosed pulmonary TB (PTB) cases, primary multi-drug resistance (MDR) and extensively drug resistance (XDR) were reported [36]. Prevalence of primary drug resistance serves as an epidemiological indicator to assess the success of the national TB control programme. Based on these findings, there is a need to give emphasis on appropriate screening of TB cases, effective and rational use of second line drugs for newly diagnosed MDR-TB patients to prevent the emergence of pre-XDR/XDR-TB strains.

Resistance to anti-TB drugs in *M. tuberculosis* arises as a result of spontaneous gene mutations that reduce the bacterium's susceptibility to the most commonly used anti-TB drugs[37]. Several previous studies have identified different genes that encode anti-TB drug targets and have briefly described different mechanisms of resistance both to RIF and INH [37, 38]. The genes can encode drug targets or drug metabolism mechanisms and influence the efficacy of anti-TB treatment [13, 39, 40]. INH resistance appears more complex and has been associated with multiple genes, most commonly *katG* and the promoter region of the *inhA* gene [27]. In the current study, we report that the most prevalent INH conferring mutation was *KatG.*Ser315Thr [9 (37.5%)]. Other studies have also shown that molecular diagnostic tests for INH resistance rely on detection of the ‘canonical’ mutations in codon 315 of *katG* and position 15 in the *inhA* promoter region. Also, many earlier studies have identified highly variable frequencies of these mutations, with *katG*315 mutations accounting for 42–95% and *inhA*–15 mutations accounting for 6–43% of phenotypic INH resistance [38, 40]. Reta and colleagues [27] found a prevalence of 95.8% for the katG315 mutation and 5.9% for the inhA promoter area mutation in patients with INH-resistant M. tuberculosis in a systematic evaluation of gene variants related with RIF and INH resistant M. tuberculosis in Ethiopia.

According to the World Health Organization (WHO), Next- Generation Sequencing is an important technique for drug-resistant tuberculosis (TB) (DR-TB) surveillance [41]. Whole Genome Sequencing offers more accurate and complete results for both first-line and second-line anti-TB medications, as well as useful insights into molecular epidemiology, such as phylogenetics, strain evolution, and transmission, than the traditional phenotypic drug susceptibility test (DST) [41]. Despite the fact that our study did not set out to compare the performance of conventional phenotypic DST and WGS, we found higher levels of MDR-TB and resistance to one or more commonly used first-line anti-TB drugs than those found in Tanzania's first national anti-TB drug resistance survey and the main survey from which the current isolates were derived. Other studies (not including national anti-TB surveys) [7, 32] have found that WGS testing of anti-TB drugs has the potential to provide comprehensive resistance detection much faster, with improved turnaround times, allowing for prompt appropriate treatment and associated patient and health-care benefits. [33].

Our study was limited to a small sample size, therefore findings of the phylogenetic distribution and association between lineages with patient demographic characteristics and drug resistance patterns may not be representative of the entire country profile. Furthermore, unavailability of data from conventional phenotypic DST methods in this study still limits our current understanding of the comparison of such methods with next generation sequencing approaches such as WGS in this setting.

## Conclusion

The findings in this study shows existence of *M. tuberculosis* strains resistant to some second line drugs which were not routinely used to treat TB in Tanzania. Lineage 3 was the most prevalent among previously treated TB cases and in TB patients living with HIV. Lineage 1 and 4 were found to be prevalent in cases that were resistant to first line anti-TB drugs. The use of next generation sequencing tools such as WGS at a national anti-TB drug resistance survey is recommended as it may improve the epidemiological findings for appropriate interventions.

## Supplementary Information

**Additional file 1: Supplementary data Table 1**. Socio demographics, clinical characteristics and drug resistance among study subjects *N*=191. **Supplementary data Table 2**. M. tuberculosis lineages and their correlation with anti-TB drug resistance, *N*=191. **Supplementary data Table 3**. Pattern of drug resistance mutations by phylogenetic lineages, *N*=24.

## Data Availability

The datasets generated and/or analysed during the current study are available at the SRA under the study BioProject ID: PRJNA807440 and at the Zenodo open access repository https://doi.org/10.5281/zenodo.6271274
