# Supplementary material for: Phylogenetic lineages of tuberculosis isolates and their association with patient demographics in Tanzania
Source: BMC Genomics. 2022 Aug 5;23:561. doi: 10.1186/s12864-022-08791-3 (PMC9356438; doi:10.1186/s12864-022-08791-3)
Supplement: Supplementary file 1 — Additional file 1: Supplementary data Table 1. Socio demographics, clinical characteristics and drug resistance among study subjects N=191. Supplementary data Table 2. M. tuberculosis lineages and their correlation with anti-TB drug resistance, N=191. Supplementary data Table 3. Pattern of drug resistance mutations by phylogenetic lineages, N=24. [file 12864_2022_8791_MOESM1_ESM.docx]

**SUPPLEMENTARY DATA**

**Supplementary data Table 1.** Socio demographics, clinical characteristics and drug resistance among study subjects N=191

| Characteristic | Frequency  n | Percentage |
| --- | --- | --- |
| Total | **191** |  |
| Sex |  |  |
| Male | 133 | 69.6 |
| Female | 58 | 30.3 |
| Age (Years) |  |  |
| Mean ± SD |  | 37.4±13.8 |
| Age group (years) |  |  |
| 0-14 | 3 | 1.6 |
| 15-24 | 29 | 15.2 |
| 25-34 | 55 | 28.8 |
| 35-44 | 52 | 27.2 |
| 45-54 | 33 | 17.3 |
| 55+ | 19 | 10.0 |
| Previous TB treatment |  |  |
| No | 172 | 90.0 |
| Yes | 19 | 10.0 |
| HIV status |  |  |
| Positive | 33 | 17.3 |
| Negative | 156 | 82.7 |
| Resistant to one or more FLD |  |  |
| Resistant | 22 | 11.5 |
| Sensitive | 169 | 88.5 |
| Resistant to all FLD |  |  |
| Resistant | 3 | 1.6 |
| Sensitive | 188 | 98.4 |
| Resistant to one or more SLD |  |  |
| Resistant | 4 | 2.1 |
| Sensitive | 187 | 97.9 |
| MDR-TB |  |  |
| Yes | 9 | 4.7 |
| No | 182 | 95.3 |

**Supplementary data Table 2:** *M. tuberculosis* lineages and their correlation with anti-TB drug resistance, N=191

| Drug resistance | Total | *M. tuberculosis* lineages | | | |  |
| --- | --- | --- | --- | --- | --- | --- |
|  |  |  |  |  |  | P-value |
| Isoniazid |  | **Beijing East Asia** | **Delhi Central Asia** | **East Africa Indian Ocean** | **European American** |  |
| Sensitive | 178 | 13 (100) | 77 (95.1) | 20 (87.0) | 68 (91.9) | 0.411 |
| Resistant | 13 | 0 (0) | 4 (4.9) | 3 (13.0) | 6 (8.1) |  |
| Rifampicin |  |  |  |  |  |  |
| Sensitive | 181 | 12 (92.3) | 76 (93.8) | 22 (95.7) | 71 (96.0) | 0.867 |
| Resistant | 10 | 1 (7.7) | 5 (6.2) | 1 (4.4) | 3 (4.1) |  |
| Ethambutol |  |  |  |  |  |  |
| Sensitive | 183 | 12 (92.3) | 79 (97.5) | 22 (95.7) | 70 (94.6) | 0.454 |
| Resistant | 8 | 1 (7.7) | 2 (2.5) | 1 (4.4) | 4 (5.4) |  |
| Pyrazinamide |  |  |  |  |  |  |
| Sensitive | 183 | 13 (100) | 81 (100) | 22 (95.7) | 67 (90.5) | 0.02 |
| Resistant | 8 | 0 (0) | 0 (0) | 1 (4.4) | 7 (9.5) |  |
| Streptomycin |  |  |  |  |  |  |
| Sensitive | 185 | 13 (100) | 81 (100) | 21 (91.3) | 70 (94.6) | 0.46 |
| Resistant | 6 | 0 (0) | 0 (0) | 2 (8.7) | 4 (5.4) |  |
| Ciprofloxacin |  |  |  |  |  |  |
| Sensitive | 189 | 12 (92.3) | 80 (98.8) | 23 (100) | 74 (100) | 0.146 |
| Resistant | 2 | 1 (7.7) | 1 (1.2) | 0 (0) | 0 (0) |  |
| Moxifloxacin |  |  |  |  |  |  |
| Sensitive | 189 | 12 (92.3) | 89 (98.8) | 23 (100) | 74 (100) | 0.146 |
| Resistant | 2 | 1 (7.7) | 1 (1.2) | 0 (0) | 0 (0) |  |
| Ofloxacin |  |  |  |  |  |  |
| Sensitive | 189 | 12 (92.3) | 80 (98.8) | 23 (100) | 74 (100) | 0.146 |
| Resistant | 2 | 1 (7.7) | 1 (1.2) | 0 (0) | 0 (0) |  |
| Ethionamide |  |  |  |  |  |  |
| Sensitive | 189 | 13 (100) | 81 (100) | 21 (91.3) | 74 (100) | 0.018 |
| Resistant | 2 | 0 (0) | 0 (0) | 2 (8.7) | 0 (0) |  |

**Note:** No resistance was reported in Amikacin, Capreomycin, kanamycin, Cycloserine, Clofazimine, PAS, Delanamid, Bedaquiline and linezolid

**Supplementary data Table 3.** Pattern of drug resistance mutations by phylogenetic lineages, N=24

| Drug-resistant mutations | Total  n (%) | Type of Lineage  n (%) | | | |
| --- | --- | --- | --- | --- | --- |
|  |  | **Lineage 2** | **Lineage 3** | **Lineage 1** | **Lineage 4** |
| Isoniazid, n=13 |  |  |  |  |  |
| CTG607CTA | 1 (7.7) | - | 1 (20.0) | 0 (0) | 0 (0) |
| Ser315Thr | 9 (69.3) | - | 4 (80.0) | 0 (0) | 5 (83.3) |
| Ser94Ala | 1 (7.7) | - | 0 (0) | 1 (50.0) | 0 (0) |
| c.-15C>T | 1 (7.7) | - | 0 (0) | 1 (50.0) | 0 (0) |
| c.-8T>A | 1 (7.7) | - | 0 (0) | 0 (0) | 1 (16.7) |
| Rifampicin, n=10 |  |  |  |  |  |
| Ser441Gln | 1 (10.0) | 0 (0) | 1 (20.0) | 0 (0) | 0 (0) |
| Gln432Glu | 3 (30.0) | 0 (0) | 0 (0) | 0 (0) | 3 (100) |
| His445Asn | 1 (10.0) | 0 (0) | 0 (0) | 1 (100) | 0 (0) |
| Leu430Pro | 1 (10.0) | 0 (0) | 1 (20.0) | 0 (0) | 0 (0) |
| Ser441Gln | 2 (20.0) | 0 (0) | 1 (20.0) | 0 (0) | 0 (0) |
| Ser450Leu | 3 (30.0) | 1 (100) | 2 (40.0) | 0 (0) | 0 (0) |
| Ethambutol, n=8 |  |  |  |  |  |
| Asp1024Asn | 1 (12.5) | 0 (0) | 1 (33.3) | - | 0 (0) |
| Gln497Arg | 2 (25.0) | 0 (0) | 1 (33.3) | - | 1 (25.0) |
| Leu359Ile | 1 (12.5) | 0 (0) | 1 (33.3) | - | 0 (0) |
| Met306Ile | 4 (50.0) | 1 (100) | 0 (0) | - | 3 (75.0) |
| Pyrazinamide, n=8 |  |  |  |  |  |
| Ala30Val | 2 (25.0) | - | - | 0 (0) | 2 (28.6) |
| E111$ | 1 (12.5) | - | - | 0 (0) | 1 (14.3) |
| GAG331TAG | 2 (25.0) | - | - | 0 (0) | 2 (28.6) |
| Leu172Pro | 1 (12.5) | - | - | 0 (0) | 1 (14.3) |
| Phe106Leu | 1 (12.5) | - | - | 1 (100) | 0 (0) |
| Thr160Ala | 1 (12.5) | - | - | 0 (0) | 1 (14.3) |
| Streptomycin, n=6 |  |  |  |  |  |
| Lys88Met | 4 (66.7) | - | - | 0 (0) | 4 (100) |
| Pro93Leu | 1 (16.7) | - | - | 1 (50.0) | 0 (0) |
| Ser172Cys | 1 (16.7) | - | - | 1 (50.0) | 0 (0) |
| Ethionamide, n=2 |  |  |  |  |  |
| Ser94Ala | 1 (50.0) | - | - | 1 (50.0) | - |
| c.-15C>T | 1 (50.0) | - | - | 1 (50.0) | - |
| Fluoroquinolones, n=2 |  |  |  |  |  |
| Ala90Val | 1 (50.0) | 1 (100) | 0 (0) | - | - |
| Asp94Gly | 1 (50.0) | 0 (0) | 1 (100) | - | - |
